# Supplementary material for: Environmental DNA concentrations are correlated with regional biomass of Atlantic cod in oceanic waters
Source: Commun Biol. 2019 Dec 10;2:461. doi: 10.1038/s42003-019-0696-8 (PMC6904555; doi:10.1038/s42003-019-0696-8)
Supplement: Supplementary file 1 — Supplementary Information [file 42003_2019_696_MOESM1_ESM.pdf]

Supplementary Figures

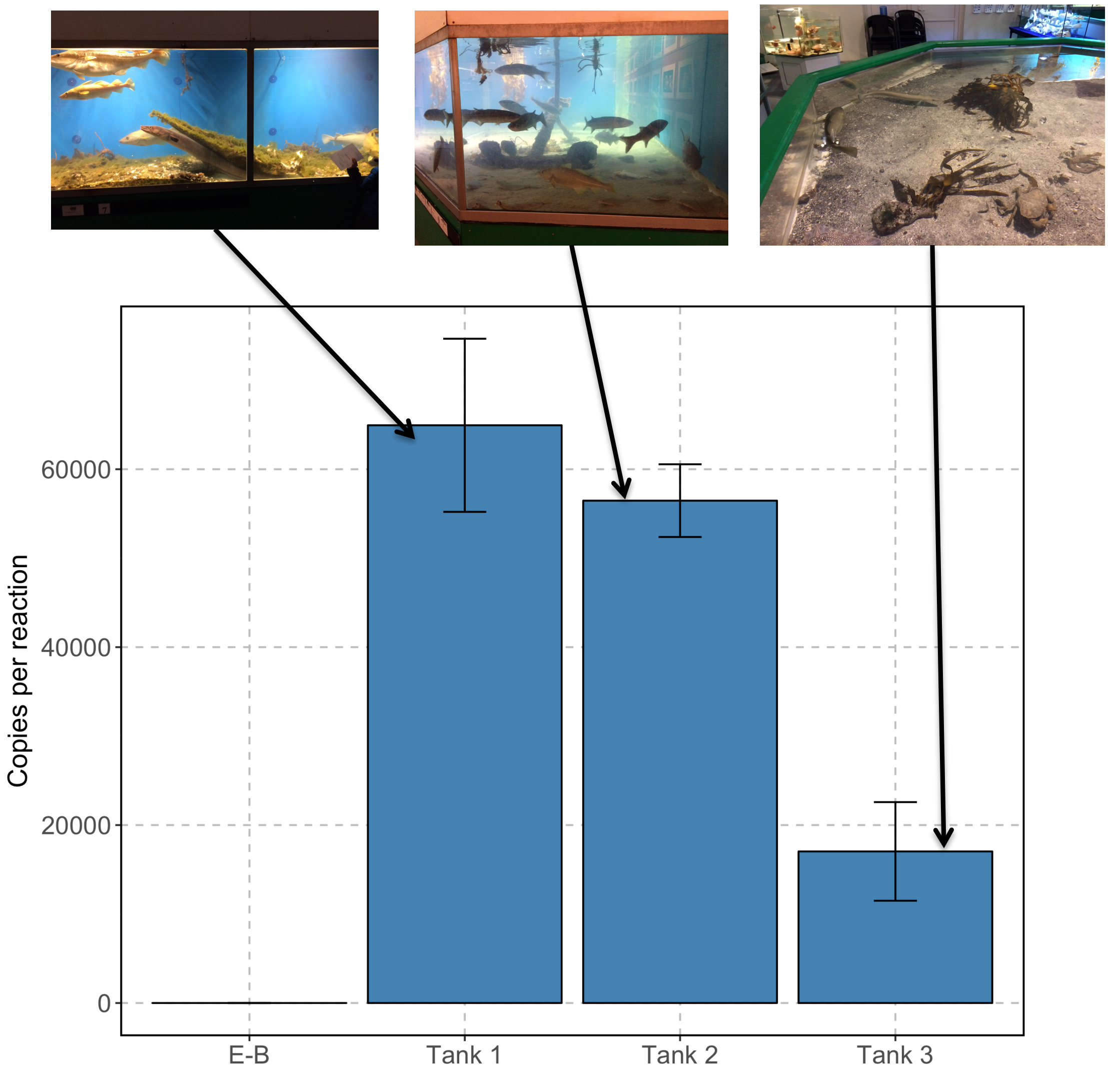

Supplementary Figure 1 - Aquarium tests. The extraction and amplification procedure (see methods) was tested in-vitro on small volume (1.5 L) water samples collected from the Faroese National Aquarium (Føroya Sjósavn). Tank 1 and 2 contained 3-5 specimens of medium-sized (40-80cm) and Tank 3 contained one specimen of a small cod (<30cm). E-B is an extraction blank of distilled water that was collected and extracted alongside the aquarium samples, it exhibited no amplification.

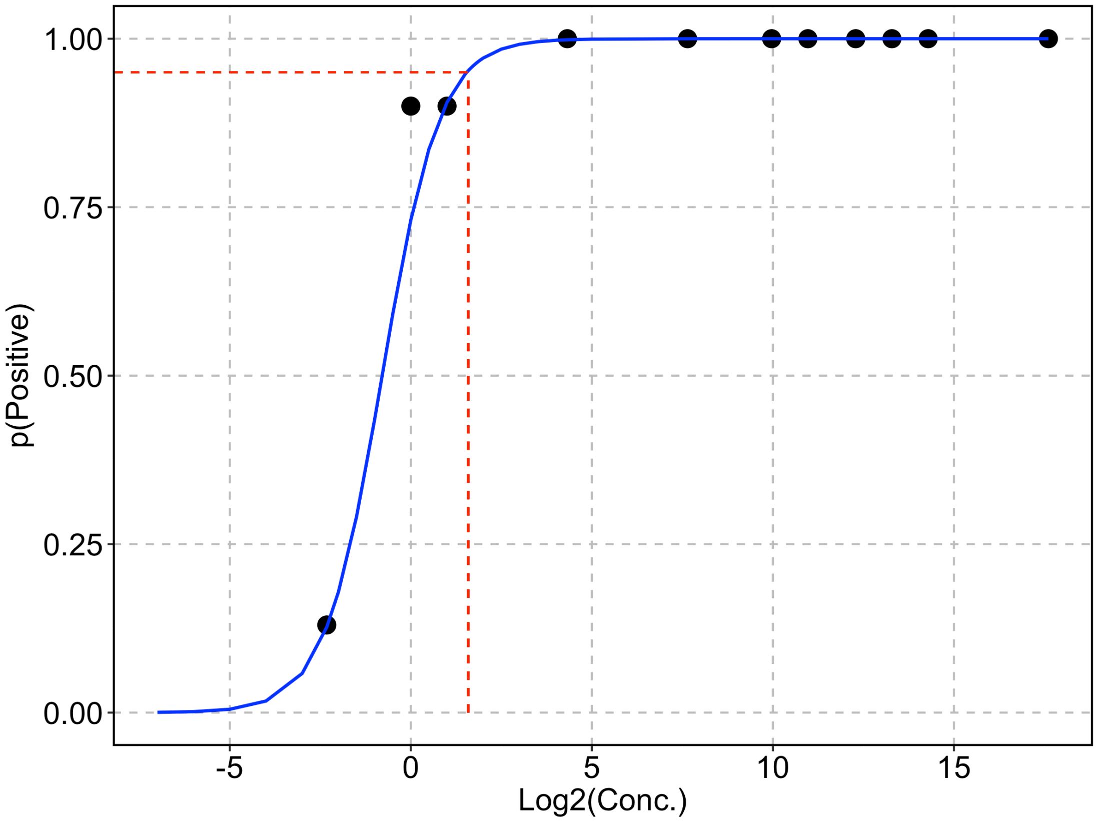

Supplementary Figure 2 - Limit of Detection. LOD was determined from a concentration dilution series. Ten replicates were amplified at concentrations of 0.2, 1, 2, 20, 200, 1000, 2000, 5000, 10000, 20000 and 200000 copies μL^{-1}. The proportion of positive amplifications is plotted against the binary logarithm of standard concentration. Logistic regression was performed using the Boltzmann Equation. LOD was determined as the minimum concentration at which 95% of the technical replicates amplified (qPCR protocol).

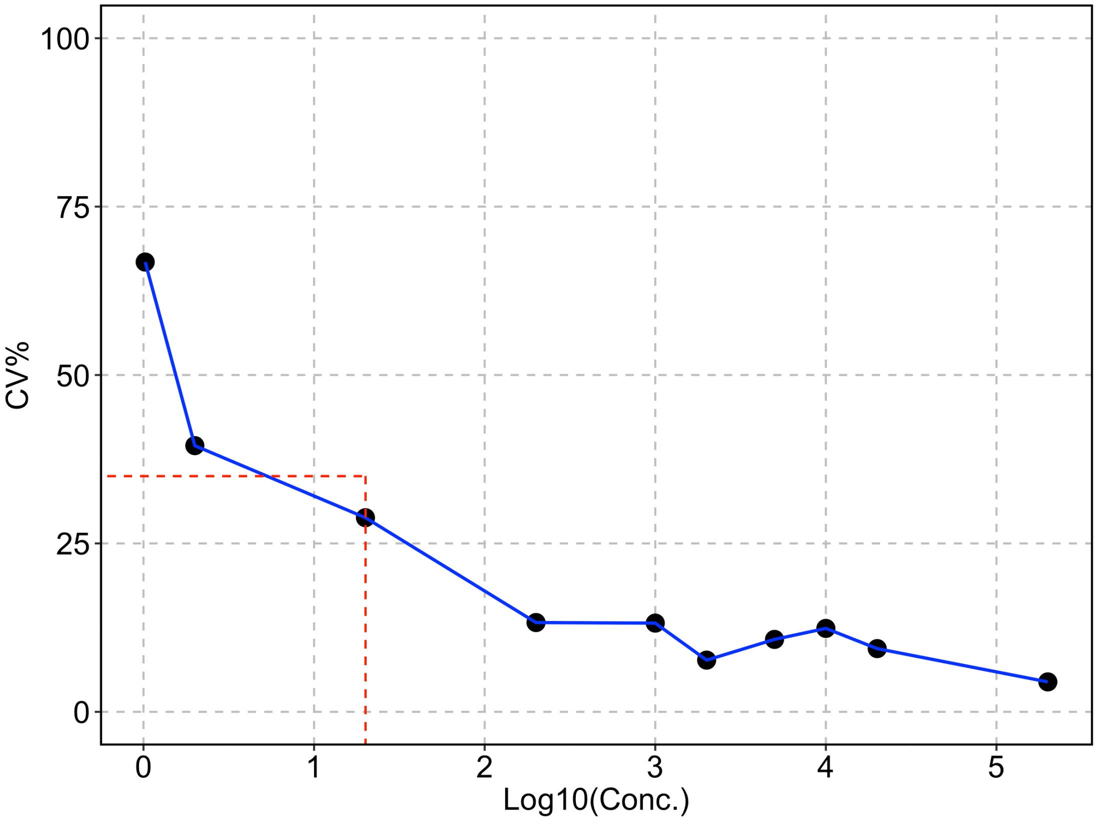

Supplementary Figure 3 - Limit of Quantification. LOQ was determined from a concentration dilution series. Ten replicates were amplified at concentrations of 0.2, 1, 2, 20, 200, 1000, 2000, 5000, 10000, 20000 and 200000 copies μL^{-1}. Standard deviation (SD) was calculated in the linear scale and expressed relative to the mean (relative standard deviation/ coefficient of variation (CV) according to the equation (CV = 100 * SD/mean). The CV was then plotted against logarithmic transformed concentration values. LOQ was calculated as the lowest concentration at where replicates show a CV ≤ 35% on back calculated concentrations[1].

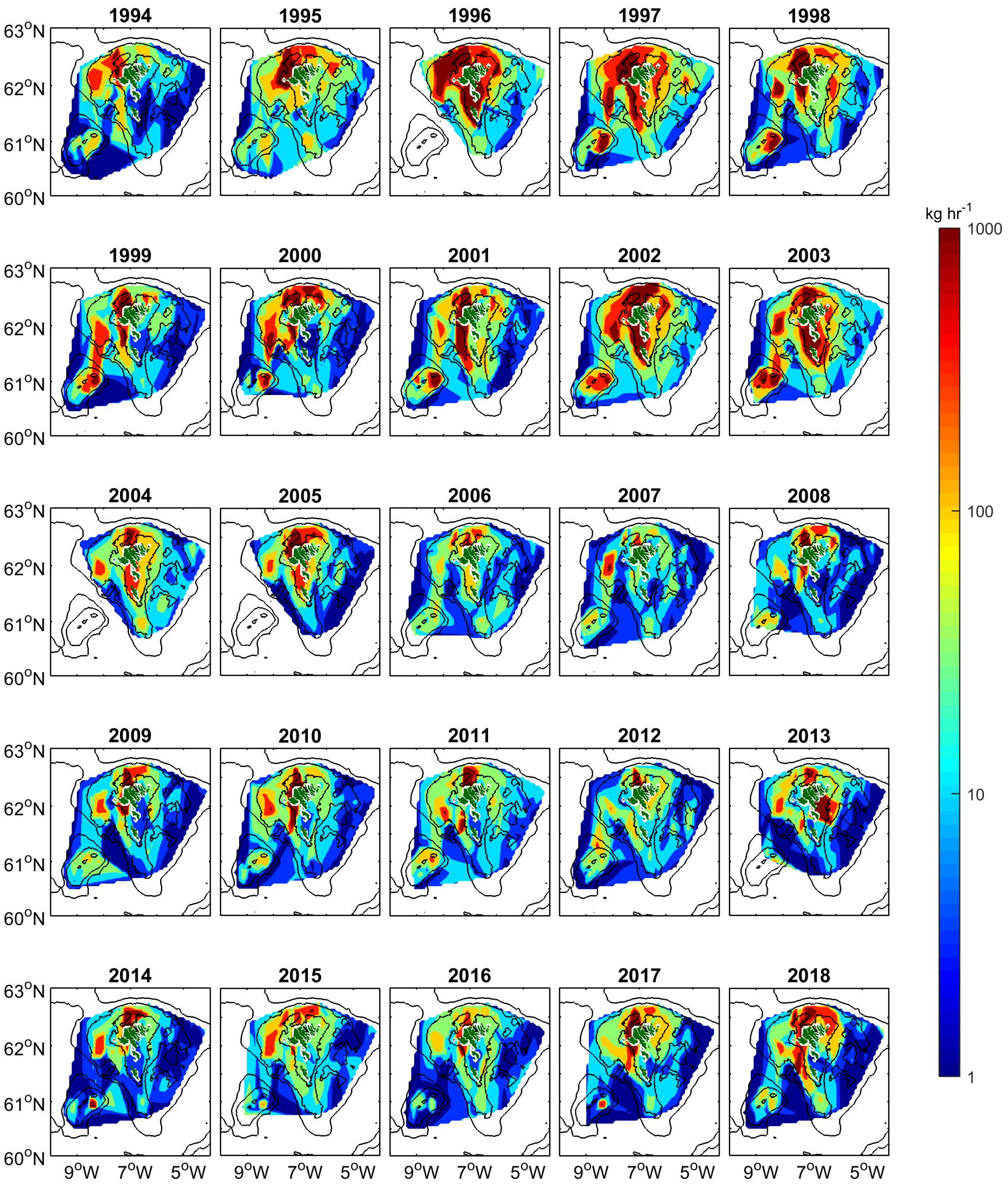

Supplementary Figure 4 - Spring demersal survey data. Annual data for Catch Per Unit Effort (CPUE) of Atlantic Cod (kg hr^{-1}) for the Faroese Shelf and Plateau Spring survey carried out by the Faroese Marine Research Institute from 1994-2018.

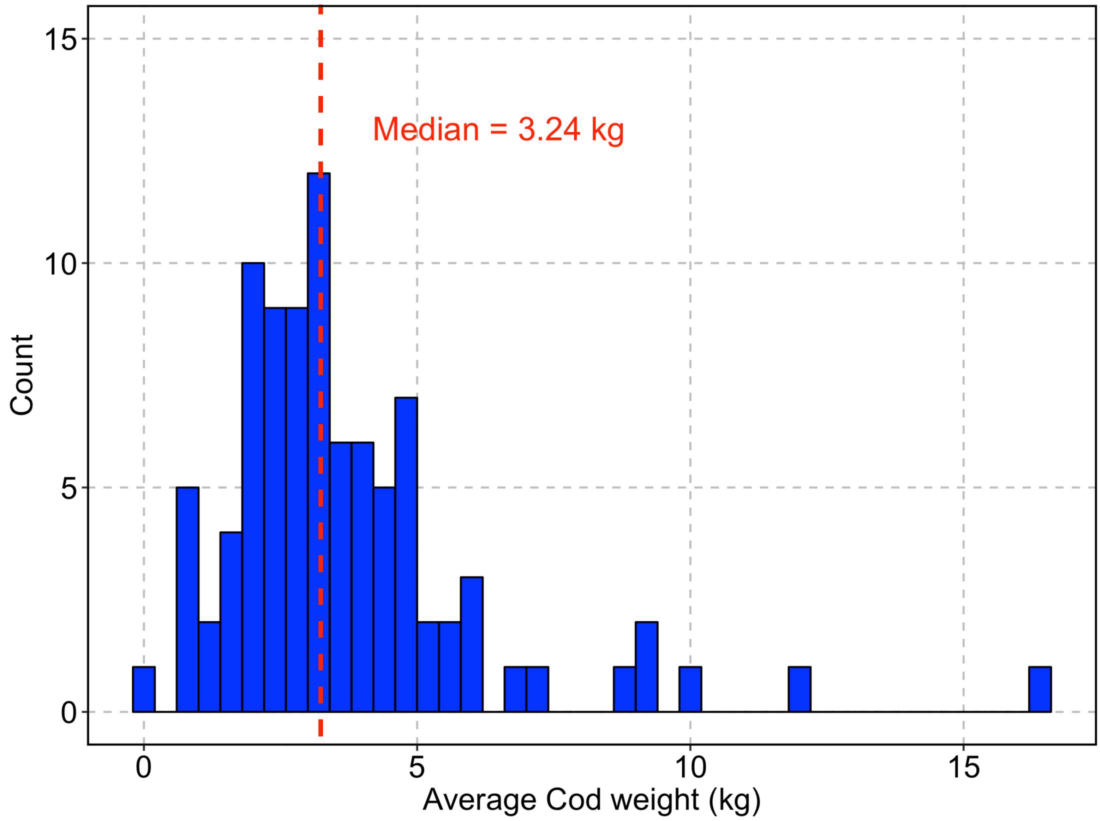

Supplementary Figure 5 - Histogram of Average Cod Size. Data are from demersal Spring Survey 2018. Bin width = 0.4 kg.

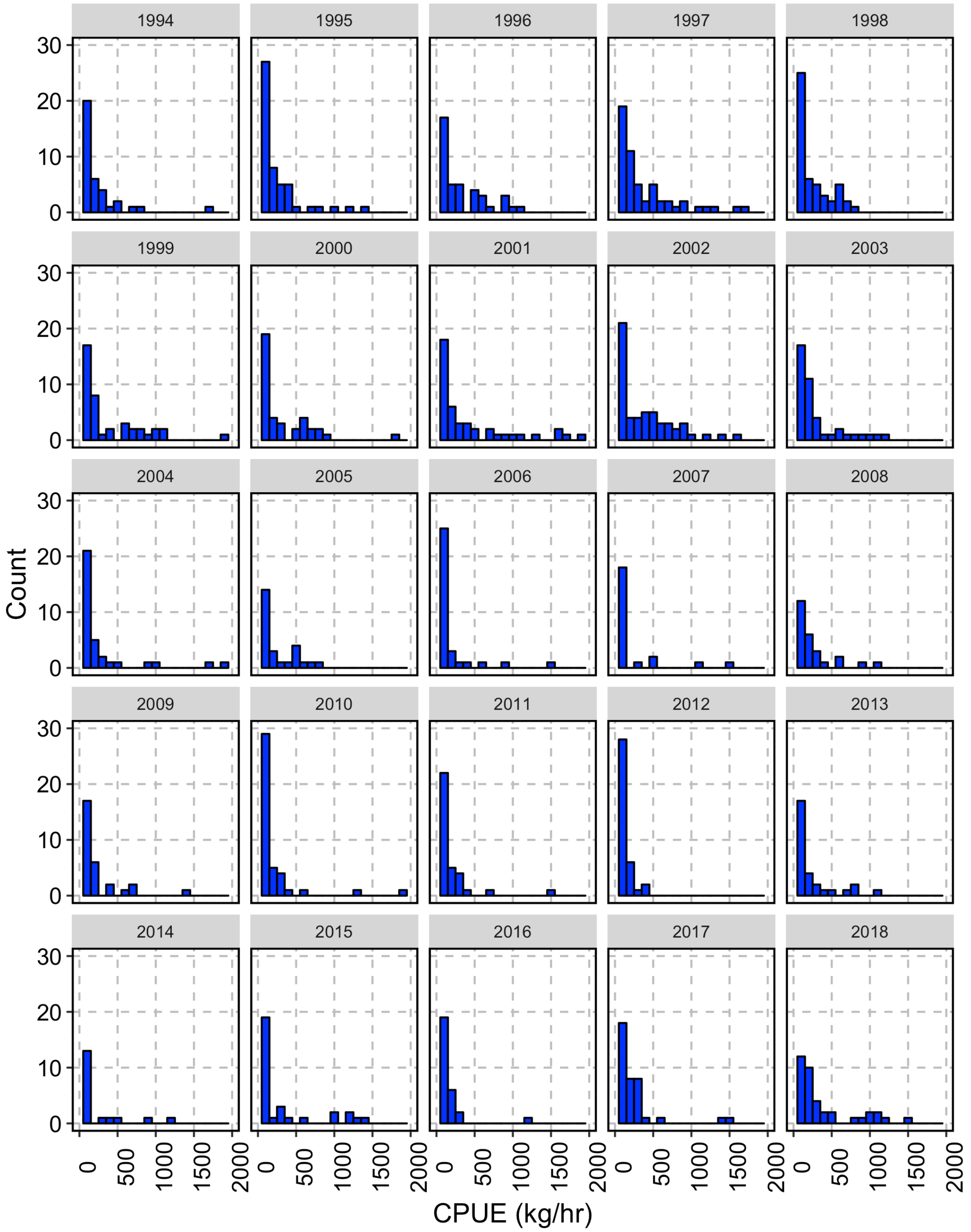

Supplementary Figure 6 - Histogram of historical CPUE data. Panels show annual frequency distributions of CPUE data for Atlantic cod caught as part of the demersal Spring survey on the Faroese Plateau and Faroe Bank. Bin width = 100 kg/hr. All years show a logarithmic distribution.

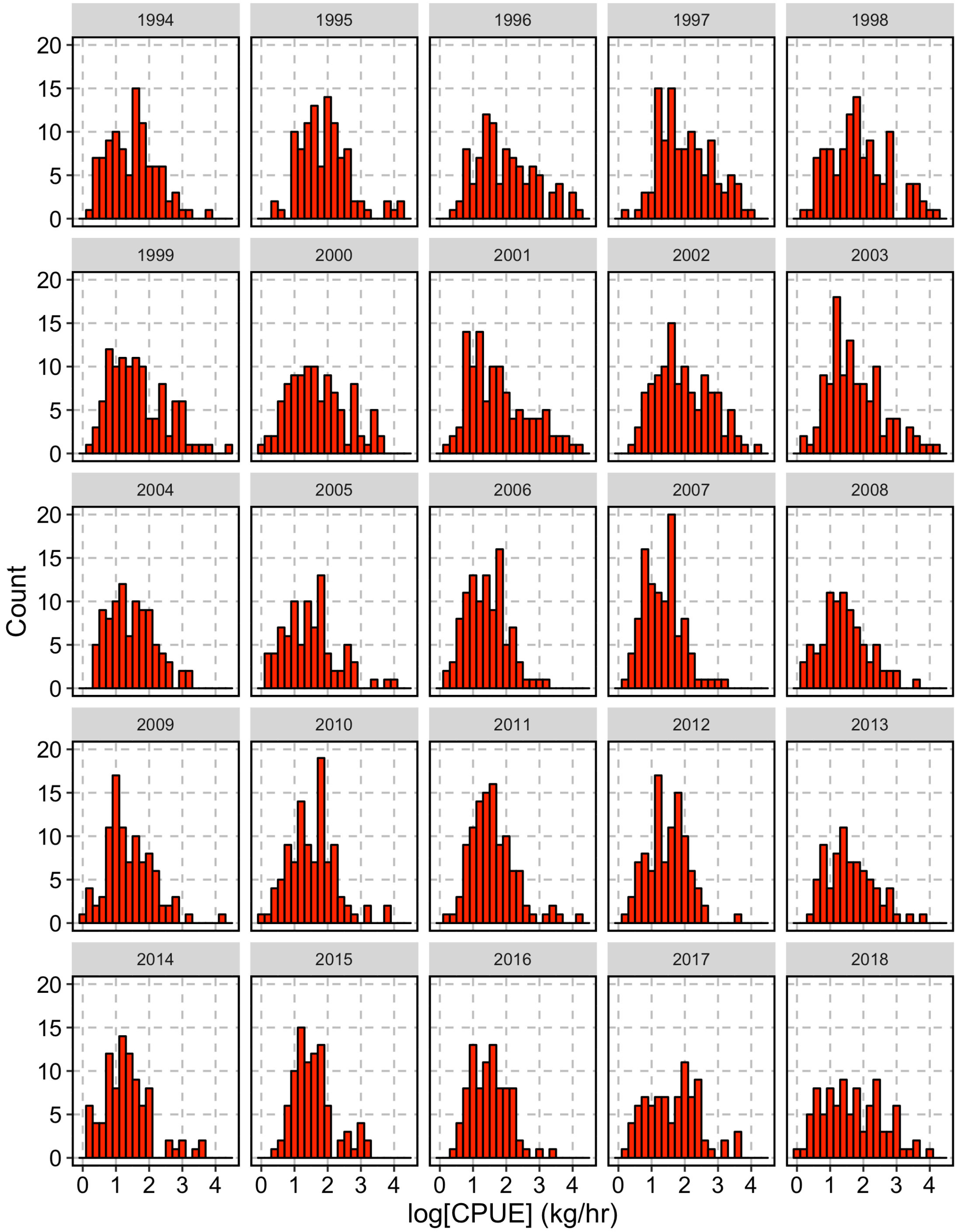

Supplementary Figure 7 - Histogram of historical CPUE data. Panels show a logarithmic transformation of frequency distribution data for Atlantic cod CPUE (Supplementary Figure 6). Bin width = 0.2.

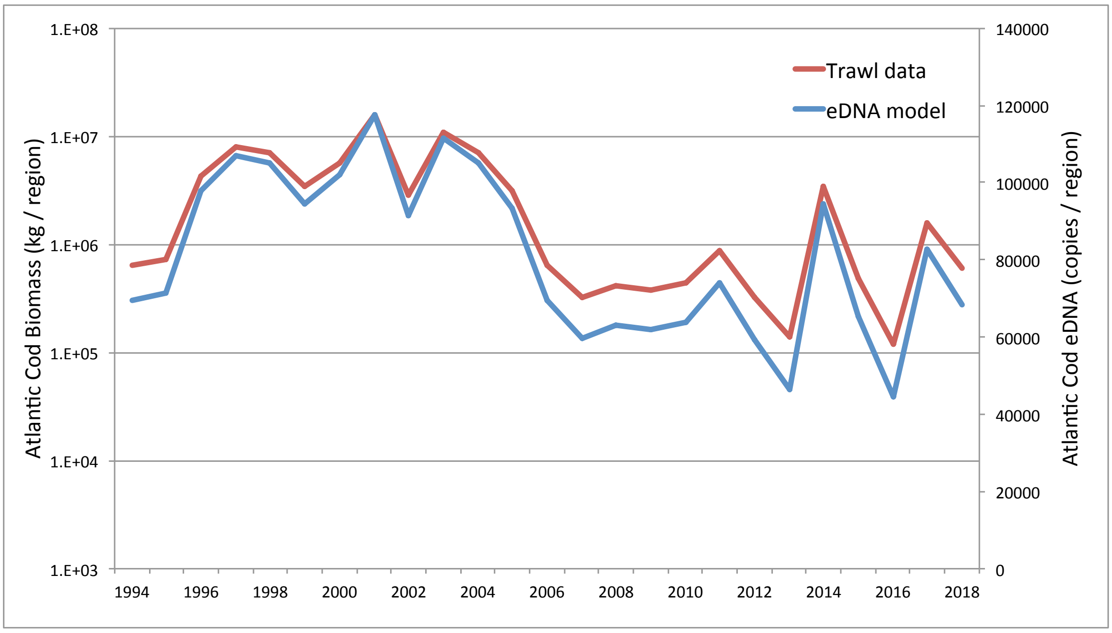

Supplementary Figure 8 - eDNA model of Faroe Bank. Panel shows a hindcast of modelled eDNA data for the Faroe Bank region based on the regression between regional biomass and regional DNA copies established in the manuscript.

Supplementary Tables

Supplementary Table 1: Biomass (kg) of closely related Gadidae species in different regions of the survey area

Region	Atlantic Cod	Haddock	Saithe	Norway Pout	Whiting	Blue Whiting	Poor Cod	Silvery Pout
**North**	2791	916	0	0	9.45	0	0.70	0
**West**	14405	838	32.3	0.54	0	0	0	0
**South**	261	384	1141	1.74	0	0	0	0
**East Coast**	734	719	73.3	0.31	124	0	0	0
**East Shelf**	21.2	582	167	635	412	0	0	10.7
**East Deep**	2.96	61.6	216	206	14	292	0	0
**Bank Edge**	18.8	170	48	1.87	1.21	20.9	7.14	0.37
**Bank Central**	596	9191	109	35.4	20	0	20.8	0

Supplementary Table 2. Summary statistics of 25-year survey data from 2018 sample regions

Region	Min	1st Quartile	Median	3rd Quartile	Max	Mean
**North**	231	905	1566	2928	5187	1936
**West**	28.1	201	703	2501	7080	1557
**South**	11.5	20.7	27.5	39.3	92.3	35.7
**East coast**	12.8	63.2	91.4	148	626	140
**East shelf**	3.17	4.66	11.0	29.5	55.9	17.0
**East deep**	0.00	1.37	3.48	5.25	68.8	7.55
**Bank edge**	0.00	1.07	5.37	14.2	80.2	13.0
**Bank central**	17.0	67.1	98.3	546	2689	478

N.B. Data are for the period 1994-2018 for the sampling positions occupied during the 2018 survey. Total catches at the stations were divided by effort (time) to calculate Catch Per Unit Effort (CPUE; kg hr^{-1}) for each region and each year. Summary statistics were then calculated based on the annual survey values for each region.

Supplementary Table 3. Sampling positions and dates for the eDNA and trawl data

Region	Trawl Station Number	Date	Time	Bottom Depth	Trawl Duration (minutes)	Lon	Lat	CTD Station Number	Lat	Lon	Date	Time
a) North	18060068	2018.03.04	09:58	104	60	-6.657	62.411	18060067	62.413	-6.661	2018.03.04	09:42
a) North	18060072	2018.03.04	16:30	109	60	-6.999	62.462	18060070	62.422	-7.129	2018.03.04	13:50
a) North	18060075	2018.03.05	08:26	100	60	-7.288	62.420	18060074	62.419	-7.282	2018.03.05	08:09
a) North	18060078	2018.03.05	12:42	104	61	-7.629	62.260	18060077	62.261	-7.624	2018.03.05	12:19
b) West	18080002	2018.03.09	06:41	111	60	-7.307	61.915	18080001	61.906	-7.308	2018.03.09	05:53
b) West	18080005	2018.03.09	09:10	124	60	-7.366	61.818	18080003	61.827	-7.359	2018.03.09	08:12
b) West	18080007	2018.03.09	11:54	130	60	-7.373	61.757	18080006	61.743	-7.364	2018.03.09	10:46
b) West	18080009	2018.03.09	16:57	127	60	-7.317	61.656	18080008	61.660	-7.317	2018.03.09	13:39
g) East deep	18060046	2018.03.01	08:28	307	60	-4.216	62.086	18060045	62.083	-4.218	2018.03.01	08:08
g) East deep	18060049	2018.03.01	13:08	278	60	-4.427	62.250	18060048	62.251	-4.426	2018.03.01	12:43
g) East deep	18060052	2018.03.01	17:07	381	61	-4.679	62.419	18060051	62.421	-4.660	2018.03.01	16:32
f) East shelf	18060037	2018.02.27	12:10	182	60	-4.909	62.001	18060036	62.002	-4.904	2018.02.27	11:49
f) East shelf	18060040	2018.02.27	17:22	163	60	-5.678	62.084	18060039	62.078	-5.676	2018.02.27	17:00
f) East shelf	18060042	2018.02.28	15:06	157	60	-5.338	62.301	18060041	62.299	-5.326	2018.02.28	14:41
f) East shelf	18060054	2018.03.02	06:38	194	60	-5.171	62.430	18060053	62.429	-5.174	2018.03.02	06:13
f) East shelf	18060030	2018.02.26	15:41	234	60	-5.643	62.187	18060029	62.179	-5.648	2018.02.26	15:18
d) East coast	18060017	2018.02.25	12:49	117	60	-6.324	61.967	18060016	61.965	-6.322	2018.02.25	12:31
d) East coast	18060020	2018.02.25	15:54	109	60	-6.150	62.088	18060019	62.086	-6.152	2018.02.25	15:30
d) East coast	18060024	2018.02.26	08:02	88	60	-6.036	62.328	18060023	62.326	-6.038	2018.02.26	07:48
d) East coast	18060027	2018.02.26	11:21	104	60	-5.986	62.415	18060026	62.416	-5.987	2018.02.26	11:05
c) South	18080017	2018.03.11	06:46	195	60	-6.181	60.920	18080016	60.921	-6.175	2018.03.11	06:17
c) South	18080019	2018.03.11	09:25	145	60	-6.517	61.012	18080018	61.011	-6.508	2018.03.11	09:00
c) South	18080021	2018.03.21	11:22	145	60	-6.660	61.097	18080020	61.096	-6.652	2018.03.11	10:58
h) Bank edge	18100040	2018.03.26	07:56	210	60	-8.286	60.846	18100041	60.918	-8.052	2018.03.26	09:48
h) Bank edge	18100044	2018.03.26	12:01	188	60	-7.982	61.002	18100043	61.003	-7.975	2018.03.26	11:38
h) Bank edge	18100045	2018.03.26	13:37	183	60	-7.967	61.078	18100046	61.116	-8.134	2018.03.26	15:00
h) Bank edge	18100028	2018.03.24	17:41	402	60	-9.370	60.484	18100027	60.519	-9.324	2018.03.24	16:45
h) Bank edge	18100031	2018.03.25	07:23	241	60	-9.034	60.499	18100032	60.565	-9.089	2018.03.25	08:37
i) Bank Central	18100002	2018.03.22	07:14	138	60	-8.191	61.171	18100001	61.169	-8.168	2018.03.22	06:53
i) Bank Central	18100006	2018.03.22	12:58	140	60	-8.668	61.156	18100005	61.115	-8.583	2018.03.22	11:59
i) Bank Central	18100014	2018.03.23	10:28	103	60	-8.662	61.018	18100013	61.018	-8.664	2018.03.23	10:14
i) Bank Central	18100017	2018.03.23	13:43	113	60	-8.349	61.020	18100016	61.034	-8.368	2018.03.23	13:15
i) Bank Central	18100019	2018.03.23	16:13	117	60	-8.376	60.984	18100018	60.987	-8.378	2018.03.23	15:56
i) Bank Central	18100035	2018.03.25	12:16	115	60	-8.922	60.716	18100034	60.715	-8.924	2018.03.25	11:40
i) Bank Central	18100037	2018.03.25	14:32	111	60	-8.937	60.840	18100036	60.833	-8.937	2018.03.25	13:41

Supplementary Table 4. Trawl and eDNA data for individual stations within regions. Positive amplifications refers to the number of technical replicates that amplified for Atlantic cod, 3/4 amplifications were required to stipulate positive amplification. Averages were calculated from the number of positive amplifications and error expressed as one standard deviation. Copies L-1 were calculated according to the methods described in the manuscript. Errors were propogated using standard formula for quadratic error propogation.

Region	Trawl Station	Date	Cod (kg hr^{-1})	Trawl detection	eDNA detection	CTD_station	Positive amplifications	Copies / reaction	±1σ	Copies L^{-1}	±1σ
a) North	18060068	2018.03.04	246.7	a) Positive	a) Positive	18060067	4	3084	754	49337	12069
a) North	18060072	2018.03.04	995.7	a) Positive	a) Positive	18060070	3	403	241	6440	3858
a) North	18060075	2018.03.05	1531	a) Positive	a) Positive	18060074	3	499	155	7989	2485
a) North	18060078	2018.03.05	17.7	a) Positive	a) Positive	18060077	4	607	431	9716	6898
b) West	18080002	2018.03.09	1199.2	a) Positive	a) Positive	18080001	4	1761	928	28174	14841
b) West	18080005	2018.03.09	4665.3	a) Positive	a) Positive	18080003	4	1692	1111	27070	17782
b) West	18080007	2018.03.09	8311.8	a) Positive	a) Positive	18080006	4	1553	251	24852	4014
b) West	18080009	2018.03.09	228.3	a) Positive	a) Positive	18080008	4	4446	1096	71136	17539
g) East deep	18060046	2018.03.01	0	c) Negative	b) Negative	18060045	1	0	0	0	0
g) East deep	18060049	2018.03.01	0	c) Negative	b) Negative	18060048	1	0	0	0	0
g) East deep	18060052	2018.03.01	3	b) <10 kg/hr	b) Negative	18060051	0	0	0	0	0
f) East shelf	18060037	2018.02.27	0	c) Negative	a) Positive	18060036	4	1820	423	29112	6764
f) East shelf	18060040	2018.02.27	1.9	b) <10 kg/hr	b) Negative	18060039	0	0	0	0	0
f) East shelf	18060042	2018.02.28	9.6	b) <10 kg/hr	b) Negative	18060041	0	0	0	0	0
f) East shelf	18060054	2018.03.02	9.7	b) <10 kg/hr	b) Negative	18060053	1	0	0	0	0
f) East shelf	18060030	2018.02.26	0	c) Negative	a) Positive	18060029	3	342	128	5467	2053
d) East coast	18060017	2018.02.25	59.5	a) Positive	a) Positive	18060016	3	337	31	5392	493
d) East coast	18060020	2018.02.25	164.7	a) Positive	b) Negative	18060019	0	0	0	0	0
d) East coast	18060024	2018.02.26	262.5	a) Positive	b) Negative	18060023	0	0	0	0	0
d) East coast	18060027	2018.02.26	247.3	a) Positive	a) Positive	18060026	4	1790	456	28640	7303
c) South	18080017	2018.03.11	23.3	a) Positive	a) Positive	18080016	4	3004	765	48056	12233
c) South	18080019	2018.03.11	130	a) Positive	a) Positive	18080018	3	692	380	11077	6076
c) South	18080021	2018.03.21	107.3	a) Positive	a) Positive	18080020	3	834	94	13339	1500
h) Bank edge	18100040	2018.03.26	0	c) Negative	b) Negative	18100041	0	0	0	0	0
h) Bank edge	18100044	2018.03.26	0	c) Negative	b) Negative	18100043	0	0	0	0	0
h) Bank edge	18100045	2018.03.26	8	b) <10 kg/hr	a) Positive	18100046	1	0	0	0	0
h) Bank edge	18100028	2018.03.24	0.1	c) Negative	b) Negative	18100027	1	0	0	0	0
h) Bank edge	18100031	2018.03.25	0.1	c) Negative	b) Negative	18100032	1	0	0	0	0
i) Bank Central	18100002	2018.03.22	32.5	a) Positive	a) Positive	18100001	4	1655	428	26476	6844
i) Bank Central	18100006	2018.03.22	192.6	a) Positive	a) Positive	18100005	3	575	380	9200	6075
i) Bank Central	18100014	2018.03.23	5	b) <10 kg/hr	a) Positive	18100013	3	450	224	7195	3580
i) Bank Central	18100017	2018.03.23	51.4	a) Positive	b) Negative	18100016	1	0		0	0
i) Bank Central	18100019	2018.03.23	254.5	a) Positive	a) Positive	18100018	4	506	197	8096	3149
i) Bank Central	18100035	2018.03.25	33.1	a) Positive	a) Positive	18100034	3	434	123	6949	1972
i) Bank Central	18100037	2018.03.25	26.6	a) Positive	a) Positive	18100036	4	243	117	3884	1873

Supplementary Table 5. Biomass and eDNA integrals and ranks within regions. Errors are propogated errors for region sums from the analysis of technical replicates and represent ±1 sd The number of sampling stations within each region (n) varied from 3-7 for both eDNA and trawl survey.

Region	Cod Biomass (kg) per region	Cod eDNA (copies) per region	Cod eDNA Error (copies) per region	Region Rank (Biomass)	Region Rank (eDNA)	n
North	2791	73482	14639	2	2	4
West	14405	151232	29329	1	1	4
East deep	3.00	0.00	0.00	8	8	4
East shelf	21.2	34579	7069	6	5	3
East coast	734	34032	7320	3	6	5
South	401	72472	13741	5	3	3
Bank edge	8.00	0.00	0.00	7	7	5
Bank central	596	61800	10672	4	4	7

Supplementary Methods

qPCR - Limit of detection and Limit of quantification

The limit of detection (LOD) and limit of quantification (LOQ) for the *Techne Gadus morhua* eDNA assay was determined from the analysis of a standard replicate curve[2]. We performed an 10-point dilution series with a concentration range of 2 x 10^{-1} – 2 x 10^{5} copies µL^{-1}, with 10 technical replicates at each concentration.

We defined LOD as the measured concentration that produces at least 95% positive replicates[1]. In order to determine this quantity we plotted the proportion of positive replicates against target copies (Supplementary Figure 2). A logistic regression was applied to the data using a Boltzman fit and the number of target molecules corresponding to 95% proportion of positive replicates was estimated by interpolation[1]. Using this technique we calculated an LOD for our qPCR assay of 3 copies per reaction, which corresponds to 52 copies L^{-1}, according to the equation: Copies L^{-1} = Copies per reaction x $[(E_{vol}/ R_{vol}) / S_{vol}]$, where E_{vol}, and R_{vol} are the extraction volume and PCR reaction volume (µL) and S_{vol} is the filtered sample volume (L).

We used two techniques to estimate the LOQ of our assay. The first technique establishes the LOQ at the concentration value where all ten technical replicates amplified [2,3]. In the present case, the LOQ determined using this technique corresponds to 20 copies per reaction (Supplementary Figure 2).

The second technique also uses the 10-point standard replicate curve, whereby standard deviation was calculated for the response of the ten technical replicates at each of the different concentrations. Standard deviation (SD) was calculated in the linear scale and expressed relative to the mean (relative standard deviation/ coefficient of variation (CV) according to the equation (CV = 100 * SD/mean). The CV was then plotted against the concentration values (Supplementary Figure 3). Following Forootan et al. (Ref 1), LOQ was calculated

as the lowest concentration at where replicates show a CV ≤ 35% on back calculated concentrations. Applying this principle to the Techne Gadus morhua speciation kit, we established that the lowest concentration that produces replicates with ≤ 35% is 20 copies per reaction (Supplementary Figure 3). The two methods used to determine LOQ yield identical values of 20 copies per reaction, which corresponds to 347 copies L^{-1}.

Supplementary References

1. Forootan, A. et al. *Biomol. Detect. Quantif.* **12**, 1–6 (2017).
2. Ellison, S.L.R., English, C.A., Burns, M.J. & Keer, J.T. *BMC Biotechnol.* **6**, (2006).
3. Waiblinger, H.-U., Graf, N., Broll, H., Grohmann, L. & Pietsch, K. *J. für Verbraucherschutz und Leb.* **6**, 411–417 (2011).
