# Supplementary material for: Environmental DNA concentrations are correlated with regional biomass of Atlantic cod in oceanic waters
Source: Commun Biol. 2019 Dec 10;2:461. doi: 10.1038/s42003-019-0696-8 (PMC6904555; doi:10.1038/s42003-019-0696-8)
Supplement: Supplementary file 2 — Description of Additional Supplementary Files [file 42003_2019_696_MOESM2_ESM.docx]

**Description of additional supplementary items**

Supplementary data 1: MIQE checklist describing qPCR experiments
