# Supplementary material for: Environmental DNA concentrations are correlated with regional biomass of Atlantic cod in oceanic waters
Source: Commun Biol. 2019 Dec 10;2:461. doi: 10.1038/s42003-019-0696-8 (PMC6904555; doi:10.1038/s42003-019-0696-8)
Supplement: Supplementary file 4 — Reporting Summary [file 42003_2019_696_MOESM4_ESM.pdf]

nature research

Corresponding author(s): Ian Salter

Last updated by author(s): Nov 2, 2019

Reporting Summary

Nature Research wishes to improve the reproducibility of the work that we publish. This form provides structure for consistency and transparency in reporting. For further information on Nature Research policies, see Authors & Referees and the Editorial Policy Checklist.

Statistics

For all statistical analyses, confirm that the following items are present in the figure legend, table legend, main text, or Methods section.

n/a	Confirmed	
☐	☒	The exact sample size (*n*) for each experimental group/condition, given as a discrete number and unit of measurement
☐	☒	A statement on whether measurements were taken from distinct samples or whether the same sample was measured repeatedly
☐	☒	The statistical test(s) used AND whether they are one- or two-sided<br>*Only common tests should be described solely by name; describe more complex techniques in the Methods section.*
☐	☒	A description of all covariates tested
☐	☒	A description of any assumptions or corrections, such as tests of normality and adjustment for multiple comparisons
☐	☐	A full description of the statistical parameters including central tendency (e.g. means) or other basic estimates (e.g. regression coefficient) AND variation (e.g. standard deviation) or associated estimates of uncertainty (e.g. confidence intervals)
☐	☒	For null hypothesis testing, the test statistic (e.g. *F*, *t*, *r*) with confidence intervals, effect sizes, degrees of freedom and *P* value noted<br>*Give P values as exact values whenever suitable.*
☒	☐	For Bayesian analysis, information on the choice of priors and Markov chain Monte Carlo settings
☒	☐	For hierarchical and complex designs, identification of the appropriate level for tests and full reporting of outcomes
☒	☐	Estimates of effect sizes (e.g. Cohen's *d*, Pearson's *r*), indicating how they were calculated

Our web collection on statistics for biologists contains articles on many of the points above.

Software and code

Policy information about availability of computer code

Data collection	Not applicable.
Data analysis	All data analysis was conducted in the R statistical environment.

For manuscripts utilizing custom algorithms or software that are central to the research but not yet described in published literature, software must be made available to editors/reviewers. We strongly encourage code deposition in a community repository (e.g. GitHub). See the Nature Research guidelines for submitting code & software for further information.

Data

Policy information about availability of data

All manuscripts must include a data availability statement. This statement should provide the following information, where applicable:

- Accession codes, unique identifiers, or web links for publicly available datasets
- A list of figures that have associated raw data
- A description of any restrictions on data availability

The data code that support the findings of this study are available from the corresponding author upon reasonable request.

Field-specific reporting

Please select the one below that is the best fit for your research. If you are not sure, read the appropriate sections before making your selection.

☐ Life sciences ☐ Behavioural & social sciences ☒ Ecological, evolutionary & environmental sciences

For a reference copy of the document with all sections, see nature.com/documents/nr-reporting-summary-flat.pdf

Ecological, evolutionary & environmental sciences study design

All studies must disclose on these points even when the disclosure is negative.

Study description	The study aimed to test the hypothesis the concentrations of Atlantic cod DNA in oceanic waters around the Faroe Islands were positively correlated with biomass obtained from a parallel standard trawl survey.
Research sample	The research samples used were bottom water samples collected from a Niskin bottle attached to a CTD rosette. Bottom water samples were collected at a depths of 1.4 to 8.7 metres above bottom. Trawl samples were also used to enumerate the biomass of cod in different regions. Additionally fish specimen samples were used to obtain tissue samples to test the specificity of qPCR primers used in the study.
Sampling strategy	The sample procedure was to collect paired water and trawl samples from approximately one third of the stations comprising the demersal spring trawl survey around the Faroe Islands. This is one of the first studies of its kind comparing eDNA and trawl surveys . We collected 35 stations, comprising approximately one third of the annual trawl survey. It is the most comprehensive sample collection and analysis conducted to date. Further considerations related to spatial scale are an active area of research
Data collection	Trawl survey data was collected and processed onboard the fisheries research survey vessel Magbus Heinason. Imponator II software was used to measure the length and weight of the fish captured in the trawls. This work was performed by research assistants and fisheries biologists employed at the Faroese Marine Research Institute. All data was stored informatically.
Timing and spatial scale	Samples were collected over a 5-week period starting on the 21st of February 2018 and ending on the 28th March 2018. The spatial scale of sample collection is approximately 100,000 square kilometres and positions within the survey area are those that have been occupied by the Faroe Marine Research Institute Spring demersal survey that has been repeated annually (1994-2018).
Data exclusions	No data were excluded from the analysis
Reproducibility	No experiments were conducted and so it was not necessary. The reproducibility of the measurements made on field samples comprised of measuring four technical replicates for qPCR analysis.
Randomization	The samples were allocated into groups based on sampling regions. These regions were defined a priori based on an analysis of 25 years of trawl survey data examining the spatial distribution of cod biomass in the study area. Sample positions within these broadly defined regions were collected randomly based on sampling opportunities on board the research vessel.
Blinding	Blinding is not relevant to this study as there were no test subjects involved.

Did the study involve field work? ☒ Yes ☐ No

Field work, collection and transport

Field conditions	Samples were collected from a research vessel in the North Atlantic experiencing various rainfall, wind and wave conditions. All samples used in this study were bottom waters that were a minimum of 88 and maximum of 402m below the surface and so were not directly influenced by atmospheric fluctuations for the timescale of sample collection.
Location	Samples were collected from a range of positions according to the demersal trawl survey. This ranged from -4.216 to -9.370 degrees East and 60.484 to 62.462 degrees North. Water depth ranged from 88 to 402m. The exact longitude, latitude and water depth of each station is provided in Supplementary Table 3.
Access and import/export	All samples were collected in Faroese waters and in accordance with Faroese governments and authorities. The Faroese Marine Research Institute is mandated by the government directly to carry out trawl surveys for fisheries management policy.
Disturbance	There is a degree of disturbance inherently associated with fishing trawl surveys.

Reporting for specific materials, systems and methods

We require information from authors about some types of materials, experimental systems and methods used in many studies. Here, indicate whether each material, system or method listed is relevant to your study. If you are not sure if a list item applies to your research, read the appropriate section before selecting a response.

Materials & experimental systems

n/a	Involved in the study
☒	☐ Antibodies
☒	☐ Eukaryotic cell lines
☒	☐ Palaeontology
☒	☐ Animals and other organisms
☒	☐ Human research participants
☒	☐ Clinical data

Methods

n/a	Involved in the study
☒	☐ ChIP-seq
☒	☐ Flow cytometry
☒	☐ MRI-based neuroimaging
